# The Clinical and Pathological Presentation of Thyroid Nodules in Children and the Comparison with Adult Population: Experience of a Single Institution

**DOI:** 10.1155/2016/1256189

**Published:** 2016-03-20

**Authors:** Tamas Solymosi, Gyula Lukacs Toth, Laszlo Budai, Istvan Gal

**Affiliations:** ^1^Thyroid Outpatient Department, Bugat Hospital, 6 Fenyves Street, Matrafured, Gyongyos 3232, Hungary; ^2^Department of Pathology, Bugat Hospital, Dozsa Gyorgy Street, Gyongyos 3200, Hungary; ^3^Department of Surgery, Bugat Hospital, Dozsa Gyorgy Street, Gyongyos 3200, Hungary; ^4^Department of Surgery, Robert Karoly Hospital, Lehel Street 59, Budapest 1136, Hungary

## Abstract

The clinical and pathological presentation of thyroid nodules among younger and adult patients was compared in an iodine-deficient (ID) region. Data of 3,010 consecutive patients younger than 20 years and 3,010 patients older than 20 years were compared. The proportion of nodular goiters (22.8% versus 39.3%), the ratio of surgically treated nodules (33.2% versus 15.2%), and the proportion of malignant nodules (4.3% versus 2.1%) among diseased patients differed significantly between the two groups (younger versus adult). Nine papillary and 1 medullary carcinoma were found among children, while 15 papillary, 2 follicular, 1 insular, 1 anaplastic, and 1 medullary carcinomas occurred among adults. The ratio of follicular adenoma to hyperplastic nodules (3 : 1 to 1 : 1.67), the proportion of follicular variant (77.8% versus 26.7%), T4 tumors (77.8% versus 33.3%), and tumors with lymph node metastasis (88.9% versus 66.7%) were significantly higher among younger papillary carcinoma patients. No malignancies occurred among spongiform and central type cysts. Similarly to iodine-sufficient regions, more nodules are malignant and carcinomas have a clinically more aggressive presentation in children in comparison with adult patients in ID. Taking the significantly greater proportion of adenomas and the lack of follicular carcinoma into account, a conservative approach has to be considered in follicular tumors among children.

## 1. Introduction

Thyroid nodules are commonly diagnosed in adults; however, they are rare cases in pediatrics. Depending on the iodine intake, they have been ranged from 19 to 35% in adults by ultrasonography (US) [1] and from 0.2 to 5.1% in children [2, 3]. Cancer risk of a thyroid nodule is significantly greater in children than in adults, as it ranges from 3 to 70% and 5 to 14% [2, 4–20] in regard to children and adults, respectively. Current research suggests that up to 25% of thyroid nodules in children are malignant compared to 5% in adults [2, 21]. The possibility of the wide range of cancers led in the past some experts to recommend that children with nodules should proceed directly to thyroidectomy without fine needle aspiration cytology (FNAC) or other preoperative testing. Also, thyroid cancer is present in children in a clinically more aggressive form as regards metastases and extrathyroidal extension [9, 22, 23]. The epidemiological data differs from one research group to another, likely reflecting methodological differences (e.g., tertiary pediatric centers are prone to overestimation of cancer prevalence) [15].

Most of the already published studies came from iodine-sufficient regions. Iodine deficiency has a well-known and deep effect on the prevalence of thyroid nodules, on the ratio of papillary to follicular carcinomas, and even on the diagnostic power of FNAC [24].

Therefore, we analyzed our 20 years' experience with childhood nodular goiter disease in a moderately iodine-deficient region. Although the analysis was retrospective, our evaluation process has a unique advantage: the clinical research, US, and FNAC were performed by the same investigator and the pathological analysis was performed by the same histopathologist during this entire period.

## 2. Patients and Methods

In a 20-year period from 1994 to 2014 all patients (*n* = 3,010) were enrolled in the study who were aged 20 or less (Group 1) and who entered our thyroid outpatient departments for the first time. All patients underwent US and TSH determination. If the TSH was under the normal value, FT4 and FT3 test were performed, as well. If the US was not fully intact and echonormal, anti-TPO test was performed. Patients with nodules larger than 1 cm in maximal diameter and those who presented with hypoechogenic discrete lesion at least 5 mm in maximal diameter underwent FNAC.

We compared the data of Group 1 with those of 3,010 randomly selected patients who were older than 20 (Group 2). The control patients entered our thyroid outpatient department also for the first time and the evaluation process was the same as described above.

The analysis was retrospective and all clinical examinations, US, and FNACs (both the aspiration and the microscopic analysis) were performed by the same physician (Tamas Solymosi).

We used the following categories in cytological diagnosis: benign, suspicious, malignant, and nondiagnostic. Follicular tumors were grouped among suspicious cases.

There was one difference between Group 1 and Group 2 patients. Based on our previous experiences, which are in accordance with the published data of [25, 26], spongiform and central types of mixed nodules share minimal, if any, risk being malignant in* adult* patients. Therefore, if the cytological material was deficient in giving a cytological diagnosis and the solid part of the nodule presented neither microcalcification nor irregular vascularization in the event of spongiform and central type cysts in Group 2, that is, in adult patients, we gave a common US-FNAC diagnosis as a result, “*cystic nodule with less than 1% risk of malignancy*,” and grouped these lesions among benign cases. However, these lesions were categorized as nondiagnostic cytology in Group 1 because of the limited experiences in younger patients. The histopathological analysis was performed by the same pathologist (Gyula Lukacs Toth) during the entire period.

The chi-square test was used for statistical analysis.

## 3. Results

### 3.1. Comparison of Clinical Diagnosis in Patients Older and Younger Than 20 Years (Table 1)

The proportion of intact thyroids was significantly greater in younger compared with adult patients (*p* < 0.001, *χ* ^2^ = 1314.1). The proportion of autoimmune thyroid diseases occurred significantly more frequently in younger patients than in older patients, too (77.5% (799/1031) and 59.7% (1429/2394), Group 1 and Group 2, resp., *p* < 0.001, *χ* ^2^ = 100.5). The ratio of Hashimoto's thyroiditis in euthyroid state to primary hypothyroidism was significantly higher in younger than in adult patients (1.56 (397 to 255) and 0.45 (372 to 821), Group 1 and Group 2, resp. (*p* < 0.001, *χ* ^2^ = 153.1)). The proportion of hyperthyroidism in the case of thyroid dysfunctions was significantly higher among younger patients compared with older patients (29.8% (108/363) and 17.2% (171/992), Group 1 and Group 2, resp. (*p* < 0.01, *χ* ^2^ = 9.75)).

The proportion of patients with nodular goiter was significantly lower in younger than in adult patients (22.8% (235/1031) and 39.3% (942/2394), Group 1 and Group 2, resp. (*p* < 0.01, *χ* ^2^ = 87.6)).

Ultrasound characteristics of thyroid nodules are given in Table 2.

We analyzed the occurrence of discrete lesions in euthyroid Hashimoto's patients (Table 3). Significantly more patients had discrete lesions at least 5 mm in maximal diameter in Group 2 than in Group 1 (*p* < 0.01, *χ* ^2^ = 26.8), and the ratio of true nodules was also significantly higher in Group 2 compared with Group 1 (*p* < 0.01, *χ* ^2^ = 28.1).

### 3.2. Differences in the Histopathological Diagnosis between Younger and Older Patients

The ratio of adenoma to hyperplastic nodular goiter was significantly higher in younger than in adult patients (51 to 17 and 46 to 77, Group 1 and Group 2, resp. (*p* < 0.01, *χ* ^2^ = 24.8)). Younger patients were characterized by a greater proportion of papillary carcinomas, a greater proportion of follicular variant of papillary carcinomas, and a more aggressive clinical presentation (see Table 4).

### 3.3. Cytological Diagnoses and Cytohistological Comparisons in Patients Older and Younger Than 20 Years (Tables 5 and 6)

Significantly higher proportion of nodular goiters was operated on in younger patients than in adults (33.2% (78/235) and 15.2% (143/942), Group 1 and Group 2, resp. (*p* < 0.01, *χ* ^2^ = 49.2)). This is explained by two facts. First, more younger patients were operated with benign cytology because of the decision of the patients (or their parents) compared with older patents (25% (9/36) and 9.5% (9/95), Group 1 and Group 2, resp.). Second, the proportion of patients operated on the cytological suspicion because of follicular tumor was higher among younger patients than in adults (17.9% (14/78) and 11.2% (16/143), Group 1 and Group 2, resp.). The latter was the consequence of the above-mentioned fact, the significantly greater proportion of adenomas among younger compared with adult patients. Similar proportion of histologically diagnosed adenomas and lesions other than adenomas was categorized statistically correctly in a statistical manner in younger and in adult patients. The diagnostic accuracy in the event of adenomas was significantly worse compared with other lesions 60.5% (46/76) and 94.9% (112/118), adenomas and lesions other than adenoma, respectively. The proportion of adenomas was twice in younger patient compared to adult patient. This led to worse specificity, positive predictive value, and diagnostic accuracy in younger compared with adult patients.

Regarding the preoperative US diagnosis of nodules in operated Hashimoto's patients, the US diagnosis was correct in 21/23 cases. A pseudonodule is characterized by the following features: it is multiple and hypoechogenic, is in the range of 1 to 10 mm, and presents an ill-defined, frequently puzzle-like margin. The thyroid displays other signs of the underlying autoimmune disease in most cases; it has at least a minimally hypoechogenic and/or inhomogeneous basic echostructure.

Pseudonodules were overdiagnosed as true nodules in two cases (see Table 3).

### 3.4. Cystic Nodules in Younger and Older Operated Patients (Table 7)

Two of 7 and 5 of 20 peripheral type cysts proved to be malignant (Group 1 and Group 2, resp.). The preoperative cytology was suspicious or malignant in the case of the two children. No carcinoma was found among 66 spongiform or central type mixed solid-cystic nodules. The ratio of nondiagnostic FNACs was significantly higher in Group 1 compared with Group 2 (*p* < 0.01, *χ* ^2^ = 7.74). This is explained by the significantly greater proportion of spongiform and central type cysts categorized as nondiagnostic in younger compared with adult patients (12/29 (41.4%) and 5/37 (13.5%), Group 1 and Group 2, resp. (*p* < 0.05, *χ* ^2^ = 6.60)).

## 4. Discussion

There was a striking and significant difference in the spectrum of thyroid diseases among children and adults in our study. Almost 60% of children referred for an evaluation had intact thyroids, while this proportion was only 12% in adults. Around 75% of children presented with autoimmune thyroid diseases, and 21% with thyroid nodules in diseased patients. These rates were 60% and 40% in adults, respectively.

Our results demonstrate that the spectrum of nodular goiter in children living in an iodine-deficient region does not differ from those living in an iodine-sufficient area. Significantly more nodular goiters are malignant and the clinical presentation (i.e., the ratio of nonmicrocarcinomas to microcarcinomas, the ratio of papillary carcinomas metastatic to lymph nodes to nonmetastasizing presentations, and the proportion of T4 tumors) is greater in children than in the case of adults. The high ratio of the follicular variant of papillary carcinoma once again is in accordance with the previous observations [27].

Significantly smaller proportion of nodules was proved to be malignant in our series compared with most series in the medical literature, 4.5% and 25% [2, 21], our data and data in the literature, respectively. The difference is explained by the well-known goitrous effect of iodine deficiency.

There are contradictory data in the literature regarding the spectrum of benign thyroid nodules. Some authors, similarly to us, have found that adenomas are the most frequent benign disease in childhood; however, others have found hyperplastic nodules as the dominant nonmalignant disease. The proportion of adenomas among histologically verified nodules ranges from 23.3% to 61.5% [10, 13, 21, 27, 28]. This ratio was 75% in our study which is in accordance with our previous finding: iodine deficiency increases the prevalence of follicular adenoma [24]. The proportion of adenomas to hyperplastic nodules may have a deep consequence even on the evaluation process: an adenoma cannot be cytologically distinguished from a follicular carcinoma; therefore, the greater proportion of adenomas leads to a greater concern and worse results in FNAC diagnosis. This is even more important in children: the ratio of adenoma to hyperplastic nodule was significantly higher in children compared with adults.

There is a similarly important effect on the entire evaluation process regarding the ratio of follicular carcinomas to papillary carcinomas. This ratio greatly differs among researchers. Some have found no follicular carcinoma in children [10, 13, 29, 30] which showed similar results to ours, while other groups have found a ratio of follicular to papillary carcinoma from 3 : 7 to 1 : 12 [15, 21, 31–35]. Both of the above-mentioned issues, that is, the ratio of hyperplastic nodules to adenomas and the real prevalence of follicular carcinomas, reflect one of the greatest concerns in thyroidology: the correct and reproducible histopathological interpretation of follicular lesions including follicular variant of papillary carcinoma [36–38]. We may try to compare the data of different authors; however, it is very challenging to draw unequivocal consequences from the various analyses, unless the issue with the interpretation is resolved.

In our opinion it is worthy to reconsider one of the general dogmas of the evaluation of thyroid patients. This dogma means that the diagnosis of a follicular tumor clearly leads to an operation [39]. It may lead (in our opinion it already led) to an apparent discrepancy: we state that our goal is to recognize malignant thyroid nodules, but we behave as our goal would be to recognize and to operate on benign tumors, as well. The thyroidologist needs to accept the fact that the cost of recognition of follicular carcinomas involves unnecessary surgeries in the case of a follicular adenoma. However, despite the increasing number of well-differentiated thyroid carcinomas, follicular thyroid carcinoma is getting diagnosed less and less frequently [40]. This means we have to pay a high price: 51 children with follicular adenoma were sent to have surgery without anyone gaining positive results from it. If other researchers confirm our data that follicular adenoma occurs significantly more frequently and follicular carcinoma less frequently in children, then a new approach seems to be mandatory in cytologically diagnosed follicular tumors, at least in the case of children. Hamburger and Kaplan have already suggested it in 1996 [41] and we also demonstrated it in a previous work that, instead of an immediate surgery, a regular US follow-up of follicular tumors seems to be a safe and adequate way to decrease unnecessary surgery in adult patients with a FNAC diagnosis of possible follicular tumor without atypia [42]. Our suggested approach (i.e., watchful waiting) is a proposal and differs from current guidelines and clinical practice [39, 43]. Although recent genetic methods are increasingly popular in this field, the use of these techniques in differential diagnosis of follicular tumors may be questioned until the uniform histological interpretation of follicular lesions is not ensured in our opinion.

The diagnosis of thyroid nodule in Hashimoto's thyroiditis is one of the greatest challenges in thyroidology. Differences in terminology may explain the great differences in occurrence: from 5% in this study to 31.5% [43, 44]. We have found discrete lesions of at least 5 mm in maximal diameter in Hashimoto's thyroiditis in 37.3% of children and only 8.1% of these lesions meet the criteria of a nodule in a pathological sense. Both of these proportions were significantly lower in children than in adults. Many authors define a thyroid nodule as a discrete lesion within the thyroid gland that is sonographically distinguishable from the adjacent parenchyma [39, 45–47]. We think that this definition of nodule in a* radiological* sense does not correspond to the definition of a nodule in a* pathological* sense and may lead to an unacceptable high rate of overdiagnosis of discrete lesions in Hashimoto's thyroiditis as nodular goiter with a not negligible iatrogenic psychological harm. It is well-known that Hashimoto's thyroiditis may be present in as much as 95% of cases as “micronodules” or pseudonodules [48, 49]. Although the distinction between the so-called pseudonodules and true nodules is not possible in every case [50, 51], our results demonstrate that the correct differential diagnostic of the discrete echo abnormalities is possible in more than 90% of cases by thorough analysis of the echo pattern, the clinical and immunological data.

The significantly higher proportion of adenomas explains the overall worse specificity, positive predictive value, and diagnostic accuracy in children compared with adults because the diagnostic accuracy of FNAC is significantly worse for well-known reasons in adenomas than in other lesions.

We categorized those cases differently where the FNAC was not diagnostic in the event of spongiform and central type cysts. In accordance with other researchers [25, 26] we have found that the risk of malignancy in these cysts is very low in adults. Our practice shows less than 1% (in this series 0%); therefore, we gave a common US-FNAC diagnosis of “*cystic nodule with less than 1% risk of malignancy*” in adults, when the cyst was spongiform or it presented central cystic degeneration and lacked abnormal vascularization and microcalcifications. These cases were grouped into nondiagnostic categories in children based on the limited experiences. The significantly greater proportion of nondiagnostic punctures among children may be explained by this difference in categorization. Nevertheless, other authors published even greater rates (28%) of nondiagnostic FNAC among children [10].

Despite the published data on pediatric FNAC, the sensitivity varied between 40% and 100% [34]; surprisingly great proportion of authors, similarly to us, found no false negative FNAC among children [10, 20, 29, 52–55]. This may be explained by the rare occurrence of papillary microcarcinomas among young patients [56] and the predominance of clinically more aggressive carcinomas among children which can be more easily recognized by FNAC. A lower specificity and therefore a lower positive predictive value were found among children compared either to most recent publications [29] or to our adults in our study. The difference is explained by the occurrence of adenomas which was higher in the former group than in the latter groups.

To summarize, childhood nodules are more frequently malignant and carcinomas have a clinically more aggressive clinical presentation in our iodine-deficient area, similarly to published data from iodine-sufficient regions and compared with adults. Our findings of significantly higher rate of adenomas to hyperplastic nodules and the low rate (in our series the lack of it) of follicular carcinomas among children require verification by other researchers. Nevertheless, a conservative management of certain types of follicular tumors has to be considered. Spongiform and central type cysts are very rarely malignant in children similarly to adults. If the cytological material is not sufficient to cytodiagnosis in such cysts, we prefer a combined US-FNAC diagnosis of “*cystic nodule with less than 1% risk of malignancy*” instead of a nondiagnostic cytological report; this may lessen unnecessary anxiety caused by a nondiagnostic report.

## Figures and Tables

**Table 1:** Comparison of distribution of thyroid disorders in younger and older patients.

	Group 1 (age 3–20)		Group 2 (age 21–90)		Significance
Age (min–max)	3–20		21–90		
Age (mean ± SD)	14.9 ± 3.39		47.3 ± 14.7		

	*n* ^*∗*^	%	*n* ^*∗*^	%	

All patients	3010		3010		
Intact thyroid^*∗∗*^	1730	57.5	387	12.9	
Not fully intact thyroid^*∗∗*^	249	8.3	229	7.6	
Diseased	1031	34.3	2394	79.5	
Graves' disease	108 (4)	10.5	171 (13)	7.1	
Primary hypothyroidism	255 (4)	24.7	821 (54)	34.3	
Hashimoto in euthyroid state	397 (12)	38.5	372 (50)	15.5	
Hashitoxicosis	39	3.8	65 (5)	2.7	
Congenital hypothyroidism	5	0.5	0	0.0	
De Quervain's thyroiditis	1	0.1	37	1.5	
Acute thyroiditis	0	0.0	2	0.1	
Autonomous nodule	6	0.6	45	1.9	
Other nodules	209	20.3	775	32.4	
Previously operated	11	1.1	106	4.4	

^*∗*^The number of patients with thyroid nodule greater than 1 cm in maximal diameter is given in parentheses.

^*∗∗*^These terms were used for those patients who were euthyroid and presented an echonormal thyroid on ultrasound, and the thyroid contained no discrete lesion or only discrete lesion less than 5 mm in maximal diameter, intact thyroid or not fully intact thyroid, respectively.

**Table 2:** Ultrasound characteristic of thyroid nodules.

	Group 1 (age 3–20)		Group 2 (age 21–90)		Significance
Age (min–max)	6–20		21–90		
Age (mean ± SD)	15.1 ± 3.21		47.8 ± 13.2		
Male	42	17.9	216	22.9	
Female	193	82.1	726	77.1	

	*n*	%	*n*	%	

All patients	235		942		
Patients with solitary nodule	152	64.7	331	35.1	*p* < 0.001
Patients with multiple nodules	83	35.3	611	64.9	
All nodules	327		1829		
Cystic nodule	106	32,4	518	28.3	
Hypoechogenic	173	52.9	729	39.9	*p* < 0.001
Echonormal	29	8.9	194	10.6	*p* < 0.001
Hyperechogenic	19	5.8	388	21.2	*p* < 0.001
Maximal diameter of nodules (median)	13		19		

**Table 3:** The occurrence and type of discrete lesions in euthyroid Hashimoto patients.

	Group 1 (age 3–20)			Group 2 (age 21–90)		
	Ultrasound diagnosis	Histological diagnosis		Ultrasound diagnosis	Histological diagnosis	
		No nodule	Nodule present		No nodule	Nodule present
No discrete lesion^*∗*^	249	2^*∗∗*^	0	164	2^*∗∗*^	0
Pseudonodule	136	3^*∗∗*^	0	158	5^*∗∗*^	0
True nodule	12	1	3	50	1	6
	397			372		

^*∗*^Included discrete lesions < 5 mm in maximal diameter.

^*∗∗*^Patients were operated on compression signs caused by goiter.

**Table 4:** Histological analysis of malignant tumors.

	Group 1 (age 3–20)	Group 2 (age 21–90)	Significance
All carcinomas	10	20	
Medullary carcinoma	1 (10%)	1 (5%)	
Follicular carcinoma	0	2 (10%)	
Insular carcinoma	0	1 (5%)	
Anaplastic carcinoma	0	1 (5%)	
Papillary carcinoma	9 (90%)	15 (75%)	
Follicular variant	7/9 (77.8%)	4/15 (26.7%)	*p* < 0.05
Microcarcinoma	0/9 (0%)	4/15 (26.7%)	*p* < 0.05
T4 tumor^*∗*^	7/9 (77.8%)	5/15 (33.3%)	*p* < 0.05
N1a or N1b tumor^*∗*^	8/9 (88.9%)	10/15 (66.7%)	*p* < 0.05

^*∗*^T4 tumor means a tumor of any size extending beyond the thyroid capsule to invade subcutaneous soft tissues, larynx, trachea, esophagus, or recurrent laryngeal nerve.

N1a metastases to level VI (pretracheal, paratracheal, and prelaryngeal lymph nodes).

N1b metastases to unilateral, bilateral, or contralateral cervical (level I, II, III, IV, or V) or retropharyngeal or superior mediastinal lymph nodes (level VII).

**Table 5:** Cytohistological comparison in children and in adults.

	Histological diagnosis							
	Group 1 (age 3–20)				Group 2 (age 21–90)			
Cytology	*n*	Not tumor	Adenoma	Carcinoma	*n*	Not tumor	Adenoma	Carcinoma
Benign	36	15	21	0	95	69	25	1
Suspicious, follicular tu.	14	0	14	0	16	2	12	2
Suspicious, other	5	0	2	3	8	3	2	3
Malignant	7	0	0	7	13	0	0	13
Not diagnostic	16	2	14	0	11	4	6	1
Total	78	17	51	10	143	77	46	20

**Table 6:** Comparison of statistical analysis of cytohistological comparison.

	Group 1 (age 3–20)	Group 2 (age 21–90)
Statistically correct grouping of adenomas	56.8% (21/37)	64.1% (25/39)
Statistically correct grouping of nodules other than adenomas	100% (25/25)	93.5% (87/93)
Proportion of adenomas	65.4% (51/78)	32.2% (46/43)
Sensitivity	100% (10/10)	94.7% (18/19)
Specificity	69.2% (36/52)	83.2% (94/113)
Diagnostic accuracy	74.2% (46/62)	84.8% (112/132)
Positive predictive value	38.5% (10/26)	48.6% (18/37)
Negative predictive value	69.2% (36/52)	74.3% (84/113)

**Table 7:** Analysis of surgically treated cystic nodules.

	Histological diagnosis							
	Group 1 (age 3–20)				Group 2 (age 21–90)			
	*n*	Not tumor	Adenoma	Carcinoma	*n*	Not tumor	Adenoma	Carcinoma
All nodules	78	17	51	10	143	77	46	20
Cystic nodules	36	10	24	2	57	29	23	5
Spongiform type cyst	10	5 (2^*∗*^)	5 (2^*∗*^)	0	12	8 (1^*∗*^)	4	0
Central type cyst	19	3 (1^*∗*^)	16 (7^*∗*^)	0	25	9 (2^*∗*^)	16 (2^*∗*^)	0
Peripheral type cyst	7	2 (1^*∗*^)	3 (1^*∗*^)	2	20	12 (2^*∗*^)	3	5 (1^*∗*^)

^*∗*^The number of nondiagnostic cytologies.
